# Annotations

**Published:** 1891-09-05

**Authors:** 


### Annotations.

**Sane Expenditure.**

Dr. Mercier in his book on sanity and insanity defines insanity as a state in which there is a fault in the adjusting of the organism to the environment, where the brain, although presented with a true impression, acts as if it had received a false one, and that not from the stupidity which does not comprehend what is told, but from a deeper incapacity to act in accordance with known facts, or to make those deductions from them which to the mass of mankind are most obvious. To say that all the lunatics are not shut up in asylums is a cynical half-truth scarcely worth repeating, for as soon as the disproportion between the action of a man's mind and the facts of his environment becomes markedly injurious to his fellows, some one is sure to take action to restrain his freedom. But to a large extent this is true only of things that are a public nuisance; and it is generally conceded that, short of murdering his wife and children, a man may do what he likes with his own. His house is his castle, to make a china shop or a bear-garden of, as he may please; his habits are his own beyond control, even though they are obviously suicidal in their tendencies; and his money is his own, to spend or save as he may choose. When he spends more than he possesses, incurs liabilities he can never meet, trouble may indeed ensue, but people rarely seem to think that the man who does this must be either a knave or a madman. And if he stops short of dishonest bankruptcy he may go to any extreme of penury or extravagance practically unblamed. Yet the man who, having an income of several thousands a year, saves nothing for his children, must be blind and reckless to the point of insanity. Hundreds of times this happens, and in the event of his death provident folks are sure to be called on to subscribe for the support of his widow—"who must feel the change in her position sadly, poor thing," say the collectors—and get his children into orphan asylums. If such lunatics cannot be put under the charge of some guardian who will look after their expenditure and allow them a few shillings a week for pocket money, a system of compulsory insurance should be enforced for the sake of those dependant on them. Less dangerous but more disagreeable is the man whose economy is a torture to all who come near him, who threatens to separate from his wife because she gave half-a-crown to a servant at Christmas, and lets his house fall to pieces about his ears to save the expense of repairs. He may die a millionaire, but he has lived a miserable wretch. Safe expenditure is that which keeps a true proportion between responsibilities and privileges, not stinting in comfort nor wasting in display, looking to the demands of the future without ignoring those of the present. It is not an easy thing to attain to, but money being such an essential thing, it may reasonably be assumed that he who is sane in his expenditure is not likely to be mad on any other point.

**The Vaccination Controversy.**

We publish elsewhere a letter signed "J. H.," in which our correspondent expresses disapproval of a paragraph that appeared in The Hospital some time ago, adversely criticising Mr. Alfred Russell Wallace. The Hospital has taken no side in the vaccination controversy. But there is no denying the fact that, as yet at any rate, the weight of competent authority is on the side of the vaccinators. We are not disposed to attach little importance to the labours and conclusions of Drs. Creighton and Crookshank. But neither, on the other hand, can we fail to see that such men as Dr. Bristowe and other fair-minded, capable, and experienced scientists still stand firmly by vaccination. Statistics, which, though easily manipulated by skilful persons, yet have a considerable value, are undoubtedly in favour of the vaccinators. It is true that The Hospital has opened its columns impartially to both sides. But surely this does not constitute a clai[illegible] on the part of the anti-vaccinators that their arguments alon[illegible] shall be entitled to fair editorial notice. The vaccination question is one of extreme difficulty. It is so difficult indeed that it is quite possible the twentieth century may not see the end of it. For our part we occupy the unfortunate position of the candid friend; and it is well known that the only reward of such a position is generally that of being well pounded and thumped by both sides. We have not the slightest desire to "run with the hare and hunt with the hounds." But neither will we abdicate our seat of impartial criticism for the sake of winning the enthusiastic approval of one side or the other. True science, indeed, has little to do with onesidedness and extreme views. Hundreds of years of accumulated experience, and the careful study of physiology, pathology, and sanitary science in general, will, no doubt, throw a good deal of light upon such methods of prevention and cure as have been practised by Jenner, Pasteur, and Koch. But it is quite certain that the facts and data now available are by no means sufficient to enable any man, however competent, to arrive at a thoroughly well-reasoned conclusion. The anti-vaccinators are the attacking party; and, therefore, they may be excused if they display a great deal of frothy excitement, and make flourishing displays of their weapons. But if any one of them knows so little of real science as to suppose that all the truth is on his side and all the stupidity and wilful obstinacy are on the other, he can hardly escape being self-convicted a fool. Let the vaccination controversy be intelligently carried on by all means; but let both sides clearly understand that they do not as yet know very much, scientifically, about the subject, and that, therefore, the methods of their controversy can hardly be too sober and mild.

**Alas! Poor Genius.**

Insanity is a popular subject just now, and Mr. J. F. Nisbet has published a volume on the old old theme of the insanity of genius. There is something fine in his initial generalisation: "Genius, insanity, idiocy, scrofula, gout, consumption, and the other members of the neuropathic family of disorders are so many different expressions of a common evil—an instability or want of equilibrium in the nervous system." It must be a comfort to the gouty old gourmand to know that his ailment is akin to genius; but surely Mr. Nisbet must see that his "common evil" leaves but few who cannot tell themselves that they are on the high road either to Parnassus or the lunatic asylum. Mr. Nisbet must be the tailless fox, who is quite convinced that the tails of all his brothers are expressions of a "common evil." It is easy to prove, or seem to prove, anything by instances, and we all know that Swift "began to die at the top," that Schumann ended his days in a lunatic asylum, and that Turner carried his meanness beyond all reason. Instances are like statistics, and can when judiciously manipulated prove almost anything, especially when you argue that if a poet is consumptive or a painter becomes paralysed, it is quite the same as if he were mad—all part of the "common evil." Would it not be equally just to say that these poor scrofulous, gouty, or consumptive souls were in most respects like the rest of the afflicted, but by Divine favour they were privileged to have a higher sanity than their fellows, a deeper insight into the beauty, the pathos, the mystery of life. It may be idiocy simply to jingle rhymes, or compose catching tunes, but Shakespeare and Beethoven are not reckoned as geniuses for such achievements as these, but for expressing by the respective media of verse and harmony thoughts which we who have no such power yet feel to be divine. Their power may be the result of some want of equilibrium; and a stagnant pool may throw the same reproach at a fountain, which is not only beautiful, but illustrates with its rainbow reflections an important truth in physical science. Would that such lack of equilibrium were more common. Idiots without genius are common enough.

